# Shared Delusion: Impact on the Parent-Child Relationship

**DOI:** 10.7759/cureus.67548

**Published:** 2024-08-22

**Authors:** Ngozi Adaralegbe, Ayotomide Oyelakin, Omobusayo Omotayo

**Affiliations:** 1 Department of Psychiatry and Behavioral Sciences, University of Texas Health Science Center at Houston McGovern Medical School, Houston, USA; 2 Department of Medicine, Nova Southeastern University Dr. Kiran C. Patel College of Osteopathic Medicine, Fort Lauderdale, USA

**Keywords:** shared psychosis, parent-child relationship, folie a famille, folie à deux, shared delusion disorder

## Abstract

Folie à famille, also known as shared psychotic disorder among family members, is a rare and underdiagnosed psychiatric condition. This disorder, seldom discussed in the literature, is particularly notable for its impact on clinical management and parent-child relationships. The few reported cases have predominantly focused on adult populations, leaving a significant gap in understanding how this condition manifests and affects younger individuals and family dynamics. One area that remains largely unexplored in the literature is the intersection between attachment patterns and shared psychosis, particularly in the context of parent-child relationships. Understanding this intersection is crucial, as it can provide insights into the development and perpetuation of shared delusions within families. This article presents a case study of a school-aged female with autism spectrum disorder who exhibits a multi-generational shared delusion. This unique case highlights the complexities of diagnosing and managing shared psychotic disorders in children, especially when compounded by other developmental conditions. The treatment implications are profound, requiring a careful and nuanced approach to pharmacological and psychotherapeutic interventions.

## Introduction

According to the Diagnostic Statistical Manual-5 Text Revision (DSM-5 TR), "delusions are fixed beliefs that are not amenable to change in light of conflicting evidence" [1]. The delusional contents can be in various themes, namely persecutory, religious, somatic, or grandiose. Shared delusional disorder (folie à deux, à trois, à famille) is when one person adopts a delusion from another person who already has an established delusion [1]. According to the DSM-IV, the shared delusion occurs between individuals in a close relationship and cannot be explained by other psychiatric conditions or the use of medications or substances. Although shared psychotic disorder was listed in the DSM-IV, under the DSM-5 TR, it is best classified under "Other Specified Schizophrenia Spectrum and Other Psychotic Disorders."

Studies on shared delusional disorder typically describe the individuals involved as comprising a primary "inducer" of a delusion and a secondary "induced" person who adopts the delusion. A common theme in case reports is the theory of a "strong" and "weak" personality dynamic, which governs the flow of delusions [2]. While this is generally accurate, it may also misrepresent cases where psychotic symptoms originate from a "weak" individual (e.g., a child transferring a delusion to a parent), even though this is rarely described in the literature, as the majority of shared psychosis cases described in the literature involve adults.

Given the limited time and duration of doctor-patient interactions in the clinical setting, these cases of shared delusions are often not investigated, so they can easily be overlooked and unreported, especially if the patient's safety is not immediately at risk. This often leads to under-recognition in clinical settings, with no specific prevalence reported in the literature. Common risk factors that enhance shared delusions include social isolation, speech or cognitive impairment, intellectual disability, and dependent relationships [3]. There is conflicting data regarding prevalence among different age groups and sexes, but couples, siblings, and parent-child pairs account for approximately 90% of cases [4], with other cases involving patients in close proximity to inpatient psychiatric units [5]. Additionally, risk factors for the development of shared psychosis in children and adolescents include abuse and neglect [6].

Adverse childhood experiences often impact individuals affected by shared psychosis, and they have been associated with the development of mental health conditions in adolescence and adulthood [7]. The type of attachment in child-parent relationships can also play a role in these dynamics. Disorganized attachment is linked to relational problems between children and parents [8]. However, the extent to which shared psychosis may add complexity to these relationships and impact the mental and physical health of those involved remains unstudied. The following case discussion explores other factors that contributed to the development of a shared delusion in a 10-year-old girl.

## Case presentation

We present the case of a 10-year-old female with a history of developmental delay and autism spectrum disorder (ASD). She was brought to our facility by her father for a mental health evaluation following aggressive, oppositional behaviors and self-harm that had occurred over a few hours. A few hours before the presentation, the family received news that dramatically changed their life trajectory: a court decision had awarded full custody to the father after a protracted custody dispute. While the patient was brought to our facility for assessment, the older sister was taken to another facility for management as they both had a mental breakdown when the news got to them. The entire family had initially lived together for several years until the parents separated, at which point the father had been living apart from them for about two years before the court case while the girls lived with their mother and grandmother. As per the court's decision, the patient is now required to reside with her father, while the mother has been refused visitation rights; the specific reasons behind this order are unclear to the health team.

Upon intake, the patient was a poor historian, appearing distraught and unable to fully participate due to her emotional response to the court's decision and her existing developmental delay. When asked about activities she enjoyed, she mentioned playing with her toys and her pet, which brightened her affect. However, she responded with "I don’t know" to many other questions, including those screening for manic and depressive symptoms. Collateral information from the father indicated that the patient had become aggressive and violent at home after the court ruling. The father also revealed that allegations made against him of being a "serial killer," causing harm to the patient, her sister, their mother, and their grandmother, had been investigated by the police and child protective services and found to be false. During her stay, the social worker confirmed through a reliable source that, per the court ruling, the mother does not have access to the daughters. Given the court restraining order, the team had limited ability to gather collateral information. We could not also interview the sister, as she was a minor, even though she was older, and they all lived together. The father did not report any official psychiatric diagnoses for himself or his ex-wife. Prior to her transfer to our facility, the patient was on her home medications, guanfacine ER (Intuniv) 2 mg at bedtime and risperidone 0.5 mg twice daily. A full medical workup was completed, with all results unremarkable.

During her hospital stay, the patient often referred to her father by his first name and declined to provide details of the alleged harm when asked. Before the separation, the family had lived together for several years. The patient continued to allege that her father was dangerous, claiming he sneaked up on people and killed them while they were camping, and would constantly refer to him as a "serial killer," which the team believed was a delusion given prior extensive investigation that suggested this information to be false. She stated on multiple occasions, when prompted, that she had learned this information from her mother and grandmother. Due to her developmental delay, the patient tended to think concretely and had a limited fund of knowledge. She was in a fourth-grade special education class. After assessment, she was diagnosed with ASD, intellectual and developmental disability (IDD), and parent-child relational issues. Risperidone was continued to manage rigid thoughts and irritability, while Intuniv was continued for impulsivity and aggression. Serial family therapy sessions were added to her treatment plan.

Throughout her initial hospitalization, the patient continued to state that her father was dangerous, even during visitation with him. The Intuniv dose was increased to 2 mg, and risperidone was increased to 1 mg twice daily to optimize treatment. Despite ongoing therapy sessions, she remained minimally engaged and reported intermittent nightmares. During the first family therapy session, she was easily agitated in her father's presence, but the father remained committed to the process. The sessions were held by the child psychiatrist who also has experience in therapy. Positive parenting techniques were demonstrated during the sessions, and some role play was encouraged during the sessions. Along with family therapy, the patient also participated in group behavioral therapy with her peers. Toward the end of her hospitalization, she became more involved in activities on the unit, with reduced aggression and no self-harm behaviors. Coping skills were discussed, and she expressed her needs better compared to on admission.

The patient's father was encouraged to visit her in the hospital, but she was initially hesitant to interact with him. The team's encouragement led to the father's repeated visits, but the first encounter was extremely chaotic: the patient initially refused to see her father, hid under her mattress, and refused to engage with him. She even declined the gifts he brought, including a pink blanket, despite pink being her favorite color. It took a series of visits for the patient to feel comfortable sitting in the same room with her father without throwing a tantrum.

Given the acute nature of the hospitalization, about three to four family therapy sessions were scheduled overall. Over time, she became more cooperative, willing to return home with her father, and no longer referred to him as a serial killer. Upon discharge, after about a week of acute hospitalization, the patient was agreeable to working with her father to improve their relationship, and she requested a kitten, which the father agreed to purchase for her. Positive parenting techniques demonstrated during the serial family sessions were emphasized. Further recommendations included continuous intensive family therapy to further enhance their relationship and mitigate any possible damage caused by the "serial killer" delusion shared among the patient, her sister, their mother, and their grandmother.

It remained unclear who the "inducer" and "induced" were in this case as the team could not obtain collateral information from the patient's mother or grandmother. The patient was discharged home with her father, and outpatient follow-up visits were arranged both with the psychiatrist and the therapist.

## Discussion

Parent-child relational problems

Shared delusions significantly affect clinical presentation, management, and parent-child relationships. However, the literature has yet to document how attachment patterns intersect with shared psychosis and their impact on family relationships. While there are reports on folie à trois, there is a lack of documentation on multigenerational shared delusions and their potential influence on family dynamics.

The DSM-5 includes a code for parent-child relational problems, used when a clinician seeks to address the quality of the parent-child relationship or when this relationship affects the course, prognosis, or treatment of a mental or other medical disorder [9]. Parent-child relational problems are, therefore, worth addressing in clinical settings.

Ainsworth [10] proposed that early attachment patterns to parents or primary caregivers can influence child-parent relationships even beyond infancy. While some children develop secure attachment patterns and are more adaptable to environmental changes, others develop insecure attachment patterns that are linked to internalizing and externalizing disorders. In cases of attachment disorganization, a child exhibits disoriented or frightened behavior toward the caregiver [8]. This form of attachment is strongly associated with the development of mental health disorders across the lifespan [8].

Children tend to build upon their relationship expectations based on prior relationships within their family in the early years. It is difficult to ascertain what kind of attachment existed when the children lived with both parents together in their early years of development. No formal attachment assessment was performed on the child during presentation; however, given the situation at the time of presentation, the child exhibited features suggestive of disorganized attachment with the father. The situation was further complicated by the strained parent-child relationship resulting from the shared delusion and the father's initial absence from the family for about two years following his separation from their biological mother. During that time, the two siblings lived with their mother and grandmother, where the delusion of the father being a serial killer was reinforced, exacerbating an already strained relationship. The patient was never formally assessed for cognitive delay, so this aspect of her history remains unknown. Additionally, a possible language delay may have contributed to the persistence of the delusion, further impacting their relationship.

Although the concept of "folie à famille" has been described in the literature, this case stands out as it involved delusions spread across three individuals over three generations. When asked about the source of her information regarding her father being a serial killer, the patient often mentioned her mother and grandmother. However, due to the limited access to the mother and grandmother, the clinical team faced challenges in obtaining complete collateral information.

Family therapy

According to Carr [11], family-based interventions are instrumental in managing attachment problems. Several approaches have been found to be effective, including video feedback intervention [12] and child-parent psychotherapy [13]. In the case discussed above, child-parent psychotherapy was administered to facilitate positive parenting and improve the relationship between the patient and her father. This approach typically involves a series of sessions, with the level of intensity depending on the family's vulnerability. Highly vulnerable families often require more intensive sessions. The patient, in this case, participated in only a few sessions before her discharge, with a strong recommendation to continue therapy post-discharge. This continuation was emphasized due to the previously strained relationship between the patient and her father. Systematic literature reviews and meta-analyses [11,14] have documented the effectiveness of parent training in reducing behavioral problems in children and adolescents, as observed in this case. Despite the added complexity of ASD and developmental delay, parent training programs have been successful in individuals with co-existing neurodevelopmental conditions [11].

Various types of separation, such as physical and psychological separation, have been identified as effective ways to manage shared delusional disorder [15]. This case involved a different approach to therapy. Physical separations might not always be feasible, as noted in the literature, due to factors such as homelessness or interdependency among family members, especially when resources are limited [15]. In this case, family therapy was used to reestablish the relationship between the patient and her father.

Family therapy has been used to manage various psychiatric conditions such as obsessive-compulsive disorder and depression. However, this is the first case report documenting the use of physical separation (in this case, court-ordered) and family therapy in addressing shared delusions, with notable clinical improvement both in the patient's mental health status and in the child-parent relationship. These results highlight the potential of integrated therapeutic approaches to produce significant positive outcomes in complex cases involving shared delusions.

The limitations in the case include not obtaining collateral information from the mother and grandmother, which could have provided more robust data to support the diagnosis of a shared delusion, as the team inferred this based on the interview with the patient. Additionally, the sister, who was also on the spectrum, could have provided us with more collateral; however, given that she was a minor and was being managed at a different facility also made this more challenging. Multiple differential diagnoses could be considered, given the events preceding her presentation; however, the team decided to isolate the shared delusion.

Treatment implications/recommendations

To achieve better outcomes for patients and their families, clinicians should apply an individualized biopsychosocial framework to each case [16]. This requires identifying pre-existing clinical conditions in affected patients. In this case, for example, the concreteness and impaired cognition associated with ASD and IDD necessitated a tailored approach to therapy. An individualized treatment plan may involve offering an increased level of therapy intensity or a higher number of sessions to meet the level of vulnerability in the affected family, such as the one discussed in the case presentation.

It is crucial to understand the cultural aspects of the patient and consider how these factors might impede or facilitate treatment [17]. In cases where environmental or sociocultural circumstances do not allow for physical separation among individuals with shared delusions, psychological separation can be an alternative. This might include exposing them to new social circles, as social isolation has been identified as a factor that can foster shared delusions.

An integrated approach should be taken to ensure that key personnel in the patient's environment are included in the treatment plan [16]. Parents should be encouraged to communicate with school counselors as needed to ensure appropriate accommodations for their children. The role of social workers and case management workers cannot be overemphasized in coordinating these processes. These efforts should begin in the hospital before discharge, with continuity of care in place to increase the likelihood of improved mental health outcomes and global functioning.

## Conclusions

Shared delusions are rare, with most documented cases involving only two people. A few cases show the involvement of three individuals. The case presented in this report is unique because it involved four individuals across multiple generations: the patient, her older biological sister, their mother, and their grandmother. The patient had several co-existing conditions and showed improvement after dose adjustment of her existing medications and undergoing a few sessions of child-parent psychotherapy. This outcome underscores the importance of family therapy, specifically child-parent psychotherapy, and positive parenting in improving outcomes for patients with co-occurring morbidities.
